# Promising Markers in the Context of Mesenchymal Stem/Stromal Cells Subpopulations with Unique Properties

**DOI:** 10.1155/2023/1842958

**Published:** 2023-09-20

**Authors:** Agnieszka Smolinska, Aleksandra Bzinkowska, Paulina Rybkowska, Magdalena Chodkowska, Anna Sarnowska

**Affiliations:** Translational Platform for Regenerative Medicine, Mossakowski Medical Research Institute, Polish Academy of Sciences, 02-106, Warsaw, Poland

## Abstract

The heterogeneity of the mesenchymal stem/stromal cells (MSCs) population poses a challenge to researchers and clinicians, especially those observed at the population level. What is more, the lack of precise evidences regarding MSCs developmental origin even further complicate this issue. As the available evidences indicate several possible pathways of MSCs formation, this diverse origin may be reflected in the unique subsets of cells found within the MSCs population. Such populations differ in specialization degree, proliferation, and immunomodulatory properties or exhibit other additional properties such as increased angiogenesis capacity. In this review article, we attempted to identify such outstanding populations according to the specific surface antigens or intracellular markers. Described groups were characterized depending on their specialization and potential therapeutic application. The reports presented here cover a wide variety of properties found in the recent literature, which is quite scarce for many candidates mentioned in this article. Even though the collected information would allow for better targeting of specific subpopulations in regenerative medicine to increase the effectiveness of MSC-based therapies.

## 1. Introduction

Mesenchymal stem/stromal cells (MSCs) attract an interest of researchers due to their wide potential applications in medicine. Multiple therapeutic properties of MSCs are thoroughly described: differentiation capacities toward mesodermal tissues, secretion of trophic factors supporting regeneration, immunosuppression, and homing functions [1]. Many clinical trials are currently conducted to confirm the safety and efficacy of MSCs treatment [1]. However, a lot of proposed therapies did not proceed to registration in the pharmaceutical market due to small success in preclinical studies or lack of progress in later stages of clinical trials [2]. A possible explanation for the insufficient efficiency of MSCs application in human studies is suggested by several causes; heterogeneity is one of them [2, 3].

MSCs heterogeneity is explored at multiple levels; variability between cells derived from different donors, sources, and isolation methods are listed as an extrinsic causes [4–6]. Even the complexity of the tissue could affect the properties of MSCs. Although the overall characteristic remains similar, the cells from different isolation sites may differ in more specific functions such as factors secretion, differentiation potential, or immunomodulatory properties, which was described for MSC isolated from adult tissues such as bone marrow or adipose tissue [7, 8]. In terms of perinatal tissues, it should be noted that even MSC of maternal and fetal origin influenced cell characteristics, such as osteogenic potential, proliferation, and immunomodulatory properties [9–11]. The variety of culture conditions and medium compositions amplifies the heterogeneity issue—applied methods differ even between good manufacturing practice facilities [12]. Described factors could be minimized with standardization of protocols and better criteria for the choice of cell source and donor, but the internal heterogeneity poses a more complex problem to solve. The development of multiomic approaches realized that many subpopulations sharing distinct gene expression profiles might exist within one population [13]. Multicolor barcode labeling revealed that the apparently homogenous MSCs population consists of several subpopulations [14]. Heterogeneity observed *in vitro* could be a result of heterogeneity *in vivo*, caused potentially by numerous mechanisms such as phenotype plasticity, transcriptional fluctuations in gene expression, proliferation ratios of different clones, and cellular senescence [15], and probably imperative one: the diverse origin of MSCs cells.

Except the identification of MSCs in a fetal liver in 7-week human embryo [16], the earlier stages of MSCs development remain rather enigmatic. Researchers propose several possible pathways of MSC formation. Somatic lateral plate mesoderm (LPM) is suggested as a major source of MSCs cells, mainly based on the described exhibition of specific markers and differentiation directions [17]. During the development, cells located in the LPM layer as a result of the epithelial-to-mesenchymal transition (EMT), originally constituting as a homogeneous population. Then, LPM cells undergo the reverse process—the mesenchymal to epithelial transition. Many cells go through multiple rounds of EMT/MET before the acquisition of their final differentiation state, adding to the complexity of the LPM environment [17]. Part of MSCs could be derived from vascular endothelial cells through the EMT process [16]. Partial origin from another germ layer is also not excluded—neural crest, a transient structure located at the neural tube, also contributes to the MSCs' origin [16–18]. Neural crest-derived stem cells undergo EMT processes, resulting in their delamination and migration to further tissues [18]—they are found in neural and nonneural tissues—craniofacial skeleton and adipose tissue are listed as one of them [18]. Potential neuroectodermal origin could explain the observed neuronal and glial markers expression by MSCs from different sources [19, 20].

**Figure 1:**
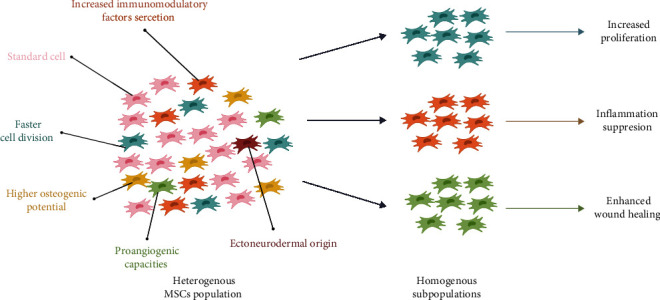
Heterogenous vs. homogenous MSCs population. MSCs population consist of distinct clones that could be separated, expanded in *in vitro* culture and applied for more specific therapeutic purposes.

The different possible origins of the cells from the heterogeneous MSCs population constitute the variety exhibited therapeutic properties such as differentiation potential, proliferation rate, secretory profile, or angiogenesis capacity (Figure 1). Among MSCs, there could be found clones showing different degrees of specialization—from line-specific progenitors to the undifferentiated stem cells that give arise into cells from all three germ layers [21, 22]. Application of selected subpopulations could increase the therapeutic efficiency and reduce observed discrepancies. However, the optimal strategy for identifying potential promising subpopulations from other morphologically similar cells still remains to be explored.

The main aim of this review is to collect available information about markers suggesting the distinct subpopulations existing within the heterogeneous MSCs population. Here, we specified two groups of markers, depending on the application: those suggesting the stem, undifferentiated character of the population, and those indicating more specified progenitors. The first group would enhance cellular therapies by providing a pool of self-renewal, proliferating stem cells. The second group could be helpful in more specific areas of regenerative medicine, such as wound healing, proangiogenesis, or anti-inflammatory agents. Expression of described markers could depend on different factors—culture condition and media composition, source of origin, donors' age and/or gender, and even more specific intercellular interactions such as a phase of the cell cycle [23]. We decided not only to compile a recent literature in this topic but also to incorporate and refresh some older evidences that could be lost and forgotten among the plethora of reports in MSCs topic.

## 2. Markers for Stem Population within MSCs

Highly heterogenous MSCs population contains subsets of cells exhibiting different stages of differentiation—stem-like cells, multipotent progenitors, and more differentiated precursors [24]. The selection of a genuine stem cell population could improve the manufacturing of MSCs. Some authors proposed even existence of pluripotent-like stem cells that would exhibit expression of characteristic genes (Oct4, Sox2, Nanog, etc.) and differentiation toward cells from all three germ layers [22]. In the topic of the search for a universal stem cell, there are still many uncertainties to be resolved, especially in the understanding of intracellular mechanisms. According to recent reports, even the phase of the cell cycle can influence the potential for cell differentiation [23]. In this section, we will focus on markers predicting the potential stem character of subpopulations. We have gathered here the information on the availability of the described subpopulations in MSC tissues (Table 1) and their observed properties (Table 2).

### 2.1. SSEA-3

Specific stage embryonic antigens (SSEA) are glycosphingolipids appearing during embryonic development. SSEA3 and SSEA4 especially aroused the researchers' interest. Both antigens occur in an earlier stage of mouse embryonic development—SSEA3 peaks in the 2–8 cell stage, while SSEA4 in the morula [56]—and then disappears [57]. For human embryos, SSEA3 and SSEA4 are detected after blastocyst formation and only in inner cell mass [57]. Although SSEA3 and SSEA4 are found on the surface of embryonic stem cells (ESCs) [58, 59] and induced pluripotent stem cells [60], their association with pluripotency is discussed. Knockout of gene B3GALT5, involved in SSEA-3/4 synthesis, drived human ESCs from primed toward naive pluripotent state [61], while iPSCs were successfully established from fibroblasts derived from SSEA3/4-depleted mice [62].

SSEA-3+ population is rather sparse within MSCs; its percentage fluctuates around 5%, depending on the sources (Table 1). SSEA-3+ cells were identified for bone marrow, adipose tissue, Wharton Jelly, and dermis [42–45, 63, 64]. Long exposition to tripsine, as well as sphere-inducing conditions, seemed to increase the SSEA-3+ cell percentage in the MSCs population [65], while a higher concentration of FBS in media decreased the SSEA-3 expression in WJ-MSCs [34].

SSEA-3+ MSCs are intensively explored in a topic of MUSE cells—multilineage differentiating stress enduring cells [22]. SSEA-3+-MUSE cells formed spheroids that were self-renewal and exhibited pluripotency—differentiated toward cells from all three germ layers upon *in vitro* culture and after *in vivo* transplantation [21, 42, 43, 66]. SSEA-3+ cells were confirmed to differentiate *in vitro* toward cells from different germ layers, such as insulin-producing cells or neural precursor cells [67, 68]. Blocking SSEA-3 in the MUSE population reduced proliferation, clonogenicity, expression of pluripotent genes SOX2 and OCT3/4, as well as differentiation toward cells from three germ layers [66]. It was suggested that SSEA-3 is involved in stemness maintenance as a coreceptor for a fibroblast growth factor (FGF-2) through the PI3K pathway [66].

The efficiency of SSEA-3+ cells was confirmed also *in vivo* in multiple animal models [52, 68–72]. SSEA-3+-MUSE cells integrated into a host tissue and differentiated into neural cells after transplantation, which facilitated neural reconstruction in an animal model of lacunar stroke [72]. A similar effect was observed in the animal model of liver fibrosis and in human liver transplantation—transplanted cells integrated into a regenerated area and spontaneously differentiated into cells associated with major liver components [52, 73]. Differentiation toward specific tissues was observed in the treatment of aneurysm and acute myocardial infarction [71, 74]. Despite different places of cell injection tested in preclinical studies, MUSE cells were identified mostly in the damaged tissues due to their unique homing capacity [52, 71]. Injection of SSEA-3+ cells was safe—no tumors nor adverse effects occurred during the long-term follow-up period [52, 72, 75]. Human trials involving patients with myocardial infarction showed the efficiency and safety of intravenous administration of MUSE cells [76]. Currently, the phase 1 clinical trial is evaluating the safety and tolerance of MUSE cells in the treatment of neonatal hypoxic encephalopathy (NCT04261335) [77].

### 2.2. SSEA-4

SSEA-4 is synthesized from SSEA-3 by ST3GAL2-enzyme *β*-galactoside *α*2,3-sialyltransferase 2 [78], and its structure contains terminal sialic acid [78]. SSEA-4+ population within MSCs is more numerous than SSEA-3, but still, different authors report different values (Table 1). SSEA-4 numbers depend on the culture medium composition [34], source of tissue [29], and donor's age [31] or gender [46]. Neonatal tissues appeared to be more abundant in SSEA-4+ cells source of MSCs than adult tissues [29] (in submission). Long-term culture as neurospheres increased the content of SSEA-4+ cells [79].

SSEA-4 was proposed as a marker to distinguish physiologically younger cells within a population obtained from elderly donors [80]. However, it is debated whether SSEA-4 is genuinely a stemness marker. Rosu-Mylers et al. [54] reported higher proliferation ratios and colony-forming capacities of SSEA-4+ cells. SSEA-4+ cells exhibited better adipogenic differentiation [33], while SSEA-4+ cells from adipose-derived MSC (AD-MSCs) generated mature endothelial cells with microvascular patterns [81]. Other authors did not observe any differences in proliferation ratio, pluripotency genes expression, and osteogenic and adipogenic differentiation between SSEA-4+ and SSEA-4− cells [34]. Interestingly, we observed increased expression of genes associated with pluripotent cells and early neuroglial cells directly after cell sorting, but it returned to the previous state after further *in vitro* culture. Similarly to He et al. [34], our group did not observe changes in proliferation and clonogenicity (in submission). So far, studies targeting SSEA-4 in MSCs population have not progressed beyond the *in vitro* phase. Despite potential embryonic origin, provided biased evidences impede classification whether SSEA-4+ subpopulation outstands from the heterogenous MSCs population.

### 2.3. CD271

CD271, also known as low-affinity nerve growth factor (NGF) receptor or p75NTR, is a neurotrophin receptor binding NGF, brain-derived neurotrophic factor (BDNF), neurotrophin-3 and 4 as well as precursors: proNGF and proBDNF [82–84]. CD271 plays a role in neurotrophins response and regulates apoptosis, cell survival, proliferation, and differentiation [84, 85]. CD271+ cells were isolated from fetal peripheral nerves [86] as well as the human adult subventricular zone [87]. CD271 is associated with neural crest-derived stem cells that migrated to different tissues during EMT [88, 89]. Interestingly, CD271 expression could be induced even earlier during development, as it was found in the murine inner cell mass of blastocyst before implantation [90].

Isolated CD271+ cells differentiated differentiation into neurons and glial cells *in vitro* and *in vivo* [86, 87]. Deletion of CD271 within sensory neurons in mice resulted in the loss of neurons that started during embryonic development and continued until adulthood [91], while CD271-depleted mice displayed a reduction in sciatic nerves and abnormal hind limb reflexes [92].

CD271+ cells were found in mesenchymal tissues, indicating a partial neural crest origin of MSCs [93, 94]. Although adult tissues contain more CD271+ cells, the subpopulation derived from fetal and neonatal tissues is more stable and decreases less rapidly with passage number than CD271+ cells in MSCs from adult tissues [30] (Table 1). Described properties of CD271+ MSCs suggest that this marker could indicate the pool of genuine stem cells that differ in embryonic origin. CD271+ MSCs differed from the rest of the heterogenous population; they proliferated more rapidly [31, 47], formed more colonies [30, 47], formed spheres, and expressed pluripotent and neural genes at higher levels [31, 48] (Table 2). Although its potential ectoneural origin, evidences for CD271+ cells from MSCs differentiation toward neurons and glial cells are lacking. Indeed, if those cells did possess this ability, they could provide an alternative source for neuron-like cells in regenerative medicine. However, according to Sowa et al. [93], a huge pool of CD271 cells found within AD-MSCs may not originate from the neural crest.

### 2.4. CD49F

The CD49f protein, also known as integrin a6 (ITG6), is a transmembrane receptor consisting of two subunits: *α* and *β*. Each subunit plays a different function, but the exact roles remain unknown. It has been found on the surface of many different stem cells' populations, such as primordial germ cells, keratinocyte stem cells, hematopoietic stem cells (HSCs), ESCs, cancer stem cells, and MSCs [95]. The principal function of the integrins family is to adhere to the extracellular matrix ligands, such as laminin, collagens, and fibronectin, and together with the tyrosine kinases, provide signals between the external and intracellular environment of the cells. The CD49f protein itself regulates the cells' interaction with the extracellular environment and mediates cell-to-cell adhesion. CD49f is considered to be a highly conservative biomarker of early-stage stem cells that is involved in their self-renewal maintenance.

The amount of CD49f marker in MSCs varies depending on the cells' source (Table 1). The highest number of CD49f-positive cells was found in BM-MSCs. Yang et al. [25] discovered that the younger the MSC cells, the higher the expression of CD49f. Their study revealed that fetal cells expressed 66.5% of the CD49f marker, while adult cells had only 11%. Moreover, during the cell culture and passage number, a gradual decrease of CD49f-positive cells was observed, from 74.8% in passage 2%–4.88% in passage 10. Interestingly, when the CD49f-positive population was sorted by fluorescence-activated cell sorting (FACS), they observed the quick loss of CD49f expression shortly after culture, from 95.2% to 48.7%. The Yang et al. [25] research also revealed better clonogenic potential of CD49f-positive cells and enhanced osteogenic differentiation. However, the influence of cytokines such as TNF-*α* on BM-MSC showed that the inflammatory environment downregulated CD49f expression, decreased adhesion ability, disturbed differentiation potential, and increased migration. In the following years, the same team successfully obtained the CD49f-positive cell population from the dermis stem cells. The isolated CD49f-high population was characterized by the presence of fibroblast markers (*Col1a1* and *Vimentin*), the ability to form spheres, and higher differentiation potential towards mesenchymal and neural lineages. Their results also provided evidence that CD49f-high cells isolated from the dermis may have neural-crest origins or may be progenitor stem cells [27].

Zha et al. [28] found that CD49f expression differed between mouse and rat AD-MSCs; mouse AD-MSCs contained 17.7% of CD49f+ cells, while rat AD-MSCs –27.2%. They also observed a gradual loss of the CD49f marker's presence during subsequent passages. After induction of the inflammatory environment and cells incubation with TNF-*α* and IFN-*γ*, a reduction in the number of CD49f markers was observed, but the cells adhesion capacity was elevated due to upregulated VCAM-1 expression. Sorted CD49f-positive AD-MSCs were characterized by increased proliferation potential, higher multilineage differentiation ability, and antiapoptotic capabilities compared to unsorted AD-MSCs.

The diversity of CD49f marker presence was also described in the work of Nieto-Nikolau et al. [26], where the different donors of bone marrow stem cells were taken under consideration. In the work of this group, they obtained 22.17%, 25.5%, and 8.65% of CD49f-positive cells isolated from different sources. Moreover, like the previous groups, they also observed a gradual loss of the marker with successive passages and similarly increased clonogenicity, migration, and differentiation potential. In addition, they found that with the lower the cell confluence expression of CD49f was higher, which may confirm the microenvironmental regulation of this integrin. They also showed that spheroid-derived MSC expressed higher amounts of CD49f together with higher proliferation, migration, and colony-forming efficiency that may be correlated, in their view, with the number of progenitor cells in MSC cultures. Earlier, another group came to similar conclusions; they proved that the sphere-forming MSC cells are rich in CD49f marker together with stemness genes expression of *NANOG*, *SOX2*, and *OCT4* compared to cells cultured in monolayer form. Therefore, CD49f may regulate the sphere-forming ability and stemness maintenance in MSC, and this regulation may be correlated with the activation of the PI3K/AKT signaling pathway [96].

In conclusion, the presented studies consistently show that the expression of the CD49f marker in MSCs is sensitive to environmental changes, such as induction of the inflammatory environment; moreover, it is also regulated by cell growth and confluence and lost during cell senescence. The results of the presented studies are consistent in terms of increased proliferation, differentiation, clonogenicity of CD49f-positive cells, and their ability to form spheres. However, the relationship between CD49f marker expression and elevated pluripotency markers for the maintenance of stemness and regulation of self-renewal needs to be thoroughly investigated.

### 2.5. GD2

GD2, a neural ganglioside, was identified in cells of the nervous system [39] and in MSCs derived from various sources: bone marrow, umbilical cord, and adipose tissue [37–40]. Its overexpression is characterized by different neuroectoderm-derived tumors related to tumor progression and metastatic potential [97].

The level of expression GD2 in MSCs fluctuated between 18% and 95%, depending on the source, culture conditions, and mouse inbred [37–40]. Martinez et al. [39] demonstrated that both BM-MSCs and AD-MSCs were characterized by the similar expression of GD2 –95%. However, the other research group reported that far less AD-MSCs expressed GD2—only 46.7%. MSCs cultured in AB-HS (human AB serum) were characterized by a higher percentage of GD2^+^ cells than MSCs cultured in FBS [37]. It's also worth noting that GD2^+^ percent in ADM-MSCs decreased from 46.7% to 31.4% or 23% when cells were cultured for 30 days in medium coming from cell culture of human glioblastoma multiforme cell line 8MGBA or A375 [38].

Sorted population of murine BM-MSCs GD2^+^ characterized higher percentage of positive cells of markers: Sca-1^+^ (stem cells antigen-1), CD105^+^, SSEA-1^+^, Nanog^+^, and lower percentage of positive cells: CD34^+^, C-kit^+^, CD45^+^, and CD11b^+^ [40]. GD2^+^ cells from both murine BM-MSCs and umbilical cord MSCs (UC-MSCs) showed higher clonogenicity, proliferation, ability to adipogenesis and osteogenesis [37, 40]. Coexpression of Sca-1^+^, CD105^+^, SSEA-1^+^, Nanog^+^, together with higher clonogenicity and proliferation by GD2+ population suggested that it could be used as a marker for early precursor cells of mouse BM-MSCs [40]. Interestingly, UC-MSCs GD2^−^ did not display a CFU-F activity [37]. However, more research concerning GD2+ cells within human MSCs are needed to confirm those observations.

### 2.6. CD349

The CD349, also known as Frizzled-9 (FZD-9), is a member family of seven transmembrane proteins that serve as receptors for Wnt proteins. CD349 is mainly expressed in pericytes, presented on the surface of capillaries and mesenchymal cells surrounding blood vessels [35]. There are also reports that CD349 is expressed on neural progenitor cells in the developing neural tube [98]. It was also shown that CD349 expression may be correlated with SSEA4, Nanog-3, Nestin, and Oct-4 upregulation in placenta-derived MSCs (PL-MSCs), and CD349-positive cells may differentiate into endoderm, mesoderm, and ectoderm cells [99]. Battula et al. [36] also showed that only about 0.2% of PL-MSCs were positive for the CD349 marker, but they had about 60-fold higher clonogenic potential. CD349 marker expression was also found by other groups, in placental decidual MSCs, which may be involved in the vascular niche building [100].

An interesting study was conducted by Tran et al. [35]. They isolated six cell lines from placental chorion leave tissue, described them by surface markers expression, and divided into groups depending on different morphology. The authors examined the CD349 expression, and in the case of two selected populations, they obtained 58% and 20% of CD349-positive cells. They also sorted one of the placental cell populations for CD349-positive and CD349-negative cells and used them in mouse vascular occlusion model experiments. They discovered that the CD349-negative cells more successfully repaired bone injury, recovered blood flow, had better effects on vessel formation, and had a greater ability of re-endothelialization in a mouse model. Interestingly, the CD349-negative population was also shown to upregulate angiogenic factors expression. Tran et al. [35], therefore, suggested that depletion of the CD349 marker may be a promising strategy in angiogenesis and arteriogenesis.

### 2.7. Sca-1

Sca-1 (stem cell antigen-1) is the mouse's surface protein of *Ly6* gene family [101]. This marker is found on adult cardiac progenitor cells or adult epicardial progenitors and is thought to be of neural crest origin [102]. Confirming these speculations on mice BM-MSC, that population PDGFR*α*^+^Sca-1^+^CD45^−^TER119^−^ expressed markers typical for neural crest such as Twist, CD271, Snail1, Snail2, Sox9 (SRY-Box transcription factor 9), and Mpz (myelin protein zero) [103]. Sca-1 was also shown for murine HSCs, mesenchymal progenitor cells, and murine BM-MSCs [41, 50, 51, 101]. Both mesenchymal progenitor cells and BM-MSCs Sca-1^+^ populations exhibited increased clonogenicity [41, 50]. Additionally, a positive population of BM-MSCs exhibited higher gene expression of *Nanog*, *TERT* (*telomerase reverse transcriptase*), *BMP2* (*bone morphogenetic protein 2*), *Myf5* (*myogenic factor 5*), decreased chondrogenesis ability and related to it decreased gene expression of *Col2a1* (*collagen type II alpha 1*). BM-MSCs from compact bone marrow expressed more Sca-1 than those derived from flushed bone marrow. BM-MSCs Sca-1^+^ cells showed an increased number of colony-forming units that exhibited greater size at 5% oxygen concentration than at 21% O_2_ concentration, which could be connected with the native location of Sca-1^+^ cells in the endosteum, where oxygen concentration is lesser. They also observed that the Sca-1 negative population of mouse MSCs did not express Sca-1 at passage 1, but due to positive cell contamination, the Sca-1^+^ cell number was increased to 90% of the population [41]. BM-MSC-Sca-1^+^ subpopulation was characterized by a lowered level of CD105, while Sca-1 expression was maintained at a high level for 22 days after sorting [51]. Despite multiple reports of Sca-1 role in murine cells, its human counterpart remains unidentified.

### 2.8. CD133

CD133 (Prominin) is characteristic of hematopoietic stem cells and neural stem cells (NSCs) and is suggested to identify cancer stem cells [104]. CD133 was found in a small subset of MSCs [29, 105]. CD133+ cells isolated from different sources expressed of some pluripotent genes than heterogenous MSCs population (mostly OCT4 and SOX2) [48]. CD133+ BM-MSCs population was suggested to secrete neuroprotection factors to treat a stroke [106]. However, CD133 properties should be wider confirmed by more research groups to clearly list it as a suitable candidate for genuine stem cell separation.

## 3. Markers for Specialized Populations within MSCs

Among the populations of MSCs, many subpopulations can be found that share characteristics with other cell types associated with other germ layers. Specific markers may support the targeting of MSCs populations into specialized cells and enhance their differentiation potential [107, 108]. In this section, we will focus on markers predicting therapeutically potential in differentiated tissue such as endothelial, neuronal, or osteocytes. The occurrence and characteristics of described subpopulations are described, respectively, in Table 3 (occurrence) and Table 4 (characteristics).

### 3.1. CD146

CD146—a melanoma cell adhesion molecule (MCAM) or surface glycoprotein MUC 18—is used as a marker for endothelial cell lineage. CD146 is a transmembrane glycoprotein constitutively expressed on vessel wall of endothelial cells, independently of the vessel type. Studies showed that CD146 was not only an MCAM but also a cellular surface receptor of numerous ligands, participating in several physiological and pathological processes [123]. CD146 intermediates many activities of various cell types such as epithelial cells, endothelial cells, macrophages, and T cells, as well as is involved in angiogenesis, development, and immune responses [127]. CD146 expression is regulated at areas of cell–cell junction, what suggests its contribution in cell–cell interaction as a mediator [128].

CD146 was detected in various many MSCs sources: bone marrow [129, 130], adipose tissue [131], umbilical cord [125, 129], synovial membrane [132], placenta, dental pulp [112, 123], and intervertebral disc [133]. Petrenko et al. [29] reported that CD146+ cells percentage was approximately in 30% of Wharton jelly MSCs (WJ-MSCs), but below 5% of BM-MSCs and AD-MSCs. In MSCs isolated from dental pulp, marker CD146 was influenced to increase proliferation, immunomodulation, and differentiation of cells. Importantly, the expression level of CD146 in MSCs from dental pulp decreased with passage after the separation [123]. Furthermore, CD146+ subpopulation from BM-MSCs was depended on oxygen levels *in vitro* due to the fact that CD146 expression was absent or very weak near the bone surface in the bone marrow niche *in situ* [130]. Localization within umbilical cord influenced the CD146 presence—the highest levels were observed in Wharton jelly. This study confirmed the potential specificity of CD146 for MSCs. Gene expression analysis revealed that. CD146 was expressed at more than three-fold higher levels in UC-MSCs compared to fibroblasts, whereas common MSC-specific markers (CD73, CD90, CD105) displayed stable expression throughout passaging [125]. The same group showed a markedly higher secretory capacity with significantly greater immunomodulatory and anti-inflammatory protein production upon inflammatory induction BM-MSCs-CD146+ compared with the BM-MSCs-CD146− [111]. Espagnolle et al. [124] showed that subpopulation CD146+ exhibited no differences in clonogenicity, proliferation, and multilineage differentiation in comparison to population CD146−, while CD146 molecule was associated with a commitment to a vascular smooth muscle cell lineage. CD146+ cells selected from human heterogenous AD-MSCs exhibited more beneficial angiogenic and adipogenic properties [110], confirming benefits in reconstructive and tissue engineering applications for AD-MSC-CD146+ cells.

Additionally, MSCs CD146+ were characterized with higher osteogenic potential—many studies revealed an association of tissue mineralization and bone reconstruction with the presence of CD146. Wrangler et al. [126] suggested that BM-MSCs CD146+ could be suitable for repopulation, whereas BM-MSCs CD146− could represent the primary choice for stimulation of endogenous intervertebral disc cells (IVDs). The CD146+ BM-MSC subpopulation possessed a greater migration potential toward degenerative IVDs, but BM-MSCs CD146− induced a stronger regenerative response. Application route (injection vs. migration) did not influence those effects Moreover, these results were independent of the application route [126]. *In vivo* murine studies defined CD146+ BM-MSCs as capable of bone formation and trans-endothelial migration [134]. Ye et al. [135] showed an association of CD146 with increased motility dependent on the Wnt signaling.

Moreover, CD146+ cells may promote mineralization and generate dental pulp-like structures, suggesting a role in self-renewal of stem cells and dental pulp regenerative therapy [112]. Interestingly, the CD146 molecule may have an impact on peripheral nerve regeneration. Shen et al. [136] suggested that CD146 not only had a key role in promoting of blood vessel regeneration but also regulated cell migration. Functional assessments showed that knockdown of CD146 decreased proliferation and viability of Schwann cells but increased their migration. Additionally, CD146 was upregulated in Schwann cells and cells associated with blood vessels following mouse peripheral nerve injury [136]. Taking together described CD146+ cells properties and its key role in vascular endothelial cell activity and angiogenesis, this subpopulation could be used in vascular smooth muscle, endothelial, or IVDs regeneration.

### 3.2. Nestin

Nestin is a class VI intermediate filament protein originally described as a marker of NSCs that is expressed during the development of the central nervous system (CNS). It is essential for stem cell survival, self-renewal, and proliferation, as well as it poses a critical regulator of cell differentiation and migration [137, 138]. *In vivo* study observation reported that NSCs cultures derived from knockout embryos showed reduced self-renewal ability, which was associated with elevated apoptosis, but no defects in cell proliferation or differentiation. In addition, nestin deficiency had no detectable effect on the integrity of the cytoskeleton [139].

MSCs Nestin+ played a key role in supporting niche activity and promote the maintenance of HSCs [140]. This population, together with PDGFR-*α* and CD51 coexpression, was characterized by fibroblastic CFUs, self-renewing capacity, and forming nonadherent mesenspheres [141]. In the other studies, the fraction of Nestin+ adult human BM-MSC expressed CD105 and CD146, which were capable of forming mesenspheres, while CD105−CD146− or CD105+CD146− cells did not generate any progeny [142]. Isern et al. [142] used transgenic mice, expressing the regulatory elements of the nestin-promotor (Nestin-GFP), to demonstrate that the MSCs Nestin+ subpopulation originated from the neural crest and had special HSCs niche functions, while the MSCs Nestin—originated from the mesoderm and gave rise to bone and cartilage. An increase in nestin expression *in vitro* was observed after MSCs culture with supplements used for neural cell culture, such as N21 or B27 [20].

Due to the intracellular localization of Nestin, numerous studies used Nestin-GFP+ transgenic mice to separate Nestin+ MSCs from various tissues, such as spleen [119], bone marrow [141–143], kidney [118], and tendon [144]. Nestin-GFP+ cells Nestin+ were isolated from kidney and expressed markers such as NG2, Sca-1, and VCAM. Those cells could differentiate into adipocytes, osteocytes, and chondrocytes under appropriate differentiation conditions. Moreover, the described population was self-renewal and exhibited high clonogenicity [118]. Huang et al. [119] found that Nes-GFP+ cells constituted about 0.68% of the total spleen cell population. Isolated Nes-GFP+ cells exhibited the characteristics of MSCs and were excellent in immunomodulation. Those observations suggested that Nestin would be used for the identification of potential markers of splenic stromal cells. Nestin+ BM-MSCs increased cell chemotaxis in myocardial infarction through paracrine activity and were involved in its regeneration [143]. TSPCs expressed higher levels of nestin than tenocytes, while isolated Nestin+ cells exhibited MSCs features, such as the capacity for colony formation and multipotential differentiation. This data suggested that nestin represented a characteristic marker of TSPCs with strong tenogenic and regenerative potential [144].

In conclusion, the expression of nestin may support the process of tendon regeneration and also affect immunomodulation. In addition, MSCs Nestin+ may maintain HSCs niche and be key in bone marrow regeneration. The problem remains the intracellular location of nestin, which limits the ability to sort human MSCs. The solution may be to find a culture method that targets MSCs to increase Nestin+ cells in a population, such as the addition of neural differentiation supplements.

### 3.3. CD200 (OX2)

CD200, also called OX2, is a membrane glycoprotein [145] responsible for the negative regulation of the number of immune cells, predominantly cells of myeloid origin [115]. That marker was expressed in different cells and tissues, such as lymphocytes and CNS [145]. Overexpression of CD200 was observed in cutaneous squamous cell carcinoma and myelodysplastic syndrome, suggesting that CD200 could also be used as a prognostic tumor marker [146, 147]. CD200-depleted mice developed faster experimental autoimmune encephalomyelitis, while binding between two CD200 receptors increased susceptibility to collagen-induced arthritis [145].

CD200 was also expressed in MSCs. According to the research from 2012, BM-MSCs had the highest level of this receptor, while it remained almost undetectable for UC-MSCs [148]. However, other research has shown BM-MSCs show about 23%–63.4% of positive cells depending on donors [114]. CD200 expression is found in AD-MSCs, but this antigen appeared to be more associated with visceral fat-derived AD-MSC (around 80% positive cells) than subcutaneous fat AD-MSC (24%) [113]. CD200 percentage for human fetal PL-MSCs was calculated as had approximately 70% of positive cells [115], while for human maternal PL-MSCs –1.8%. Additionally, fetal PL-MSC-CD200+ cells increased allograft survival in compared to maternal PL-MSCs [115]. Authors suggested that a higher number of positive CD200 cells could exaggerate better immunosuppressive effects [115]. Pontikoglou et al. [114] observed that BM MSCs CD200+ showed higher levels of *α*SM-actin (*α* smooth muscle-actin) protein, increased expression of *RUNX2* (*runt-related transcription factor 2*) and *DLX5* (*distal-less homebox 5*) *and higher* osteoblastic potential. A similar result was received by Rostovskaya et al. [149] on mice BM-MSCs. The CD200^+^ population of mouse BM-MSCs showed an increased potential of osteogenesis both at the mRNA and protein levels. In addition, they considered that CD200 could be the marker progenitor cells in osteogenesis. CD200 transfection resulted in enhanced osteogenesis and chondrogenesis of BM-MSC, as well as increased clonogenicity and stemness-related genes expression [150], which confirms the connection between CD200 and osteogenesis. CD200+ AD-MSC isolated from visceral fat exhibited reduced adipogenesis, which suggests it as a predictive marker for lowered adipogenic capacity [113]. Based on the literature, CD200 could be associated with immunogenic subpopulation as well as with osteogenic progenitors—however, this link requires further explanation.

### 3.4. VCAM (CD106)

Vascular cell adhesion molecule 1 (VCAM-1)/CD106 is a typical marker on endothelial cells and is also expressed on some stromal cells in particular vascular niches [151]. VCAM-1 is expressed on inflamed vascular endothelium, as well as on dendritic cell and macrophage-like types in both normal tissue and inflammation environment sites. VCAM-1 is important in cell–cell recognition and appears to regulate inflammation-associated vascular adhesion and the trans-endothelial migration of leukocytes, such as macrophages and T cells [152].

VCAM-1 was detected in MSCs isolated from bone marrow, umbilical cord, and placenta chronic villi (CV-MSC), while MSCs from adipose tissue were lacking this marker [120]. Yang et al. [120] proved that VCAM-1 expression on CV-MSCs was regulated in response to propagation and cytokine induction. Moreover, population CV-MSCs VCAM+ displayed a potent angiogenic property through superior angiogenic secretomes, e.g., HGF, IL-8, ANG, ANGPT2, and CXCL1 in comparison with the CV-MSC VCAM− subpopulation. As a result, VCAM+ subpopulation exerted enhanced therapeutic efficacy on regeneration after ischemia. [65]. VCAM-1 plays an important role of immunomodulation—its presence depended on inflammatory cytokines such as INF, TNF, and IL-1 [122, 153]. MSCs treated with the pro-inflammatory cytokines IFN*γ*, TNF*α*, and IL-1*β* increased the VCAM+ subpopulation to 88%. After inflammatory stimulation, VCAM+ cells still showed the capacity to multilineage differentiation potential [122]. V-CAM-1+ cells properties link this subpopulation with immune response.

### 3.5. CD142

CD142 (factor tissue) is a transmembrane protein with a little tail, which plays a key role in wound healing. The expression of this mark occurs in brain, lung, and epithelial cells of the skin, mucosa, and glomeruli, such as MSCs [154]. Sun et al. [13] observed that the percent of CD142-positive cells fluctuate between 71.2% and 88.6% in WJ-MSCs, depends on donors. Additionally, medium from culture cells CD142^+^ stimulated “would healing” in scratch test of fibroblast compared to medium from WJ-MSCs CD142^−^. WJ-MSCs CD142^+^ also shown higher gene expression of *SPARC* (*secreted protein acidic and cysteine-rich*), *COL4A1* (*collagen type IV alpha 1 chain*), *COL1A1* (*collagen type I alpha 1 chain*), *COL5A1* (*collagen type V alpha 1 chain*) and lower gene expression of *MKI67* (*marker of proliferation Ki-67*) which was related with lower proliferation. Possible procoagulant activity of CD142 may raise concern in the future *in vivo* and clinical studies. However, according to Araldi et al. [155], this is not the only universal procoagulant variable while addiction of heparin could decrease this effect.

### 3.6. CD317

Another surface antigen indicating a distinct subpopulation is CD317. Identified within BM-MSCs, CD317+ cells did not differentiate toward standard lineages: oste-, chondro, and adipocytes. CD317+ MSC exhibited increased expression and secretion of IL-7, linking this population with enhanced immunomodulatory capacity [116]. Further studies revealed that the immune profile of CD317+ MSCs contrasts with the immunosuppressive function of MSCs. CD317+ subpopulation induced Th1 proinflammatory phenotype *in vitro* as well as promoted cutaneous tissue damage *in vivo* instead of tissue formation [117]. It is suggested that CD317+ subpopulation promoted a proinflammatory response through constitutive interferon signaling [117].

## 4. Challenges in Cell Separation of Specific Subpopulations

**Figure 2:**
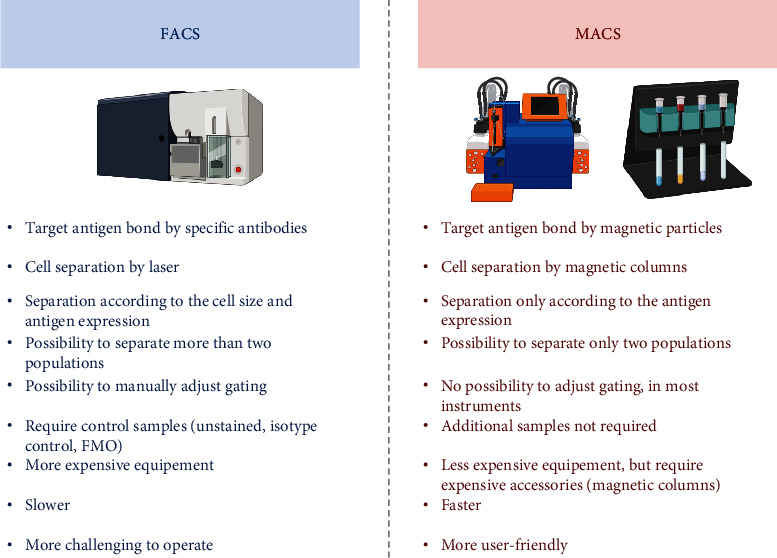
Fluorescence-activated cell sorting (FACS) and magnetic activated cell sorting (MACS)—properties comparison. Abbreviation: FMO: fluorescence minus one.

The main challenges in receiving homogenous MSC subpopulations are connected with the efficient process of cell separation. Magnetic-activated cell sorting (MACS) and FACS are the most frequently chosen methods. MACS uses microparticles to detect antigens, while the cells are separated between magnetic columns [156], while in FACS, cells are bound with fluorochrome-conjugated antibodies recognizing specific antigens and then separated with laser. The choice of selection technique is a matter of dispute, as both methods have advantages and limitations (Figure 2). According to Bowles et al. [157], MACS isolated cells with the same efficiency as FACS with reduced cell stress and increased yield. They also observed smaller contamination of unrelated cells in culture after separation, while other authors observed the opposite—FACS sorting resulted in a more homogenous population of microglia [158]. FACS is found as a less variable than MACS [159, 160] and preferred for negative selection—MACS did not sufficiently eliminate labeled cells from population, especially those exhibiting low-level expression of antigen [161]. Sutermaster and Darling [162] described that MACS and FACS outcomes were similar, but MACS required the optimization of antibody and microbead concertation. In our experiences, we observed 13 times better recovery after SSEA-4+ WJ-MSCs with FACS, comparing to MACS (in submission). Given these points, MACS works faster, requires less equipment and probes for controls, and lower machine costs. FACS allows for the selection of multiple surface antigens and also the choice of size.

The above described heterogeneity issue also should be taken into consideration in planning further experiments, especially in the context of tissue complexity. Some authors observed the differences in surface antigens expression from different compartments of the same tissue. Unfortunately, the majority of publications did not provide a specific site of tissue that was used for MSC isolation. This problem is apparent regarding perinatal tissues, e.g., “placental MSC” refers to cells isolated from different parts of the placenta: chorionic plate, chorionic villi, trophoblast or placental amniotic membrane [163, 164]. The lack of more specific information regarding the isolation site could indeed contribute to the observed disproportions in the literature and would hinder the reproduction of experimental protocols in close future.

Reaserchers report that both separation methods not always result in receiving of 100% pure fraction of positive cells. Vaculik et al. [33] scored obtaining 71% SSEA-4+ cells and 82% CD271+ cells after MACS as a great outcome. Fouad et al. [43] reported the enrichment to 82% of SSEA-3+ cells after FACS. The purity issue was observed especially during the sorting of sparse populations—MACS separation allowed to increase SSEA-3+ population from 1.9% to 77% [44]. Unfortunately, most of the scientific articles does not provide values of specific target antigen before and after separation nor calculate the efficiency of the process.

Maintenance of the homogeneity of separated populations is another problem to discuss. Many researchers observed a gradual decrease of surface antigen expression with time of cell culture for SSEA-3 [67], CD271 [55], CD49-F [25, 28], and CD146 [123]. Rosu-Myles et al. [54] observed the decrease of SSEA-4+ number during 28 days (approximately four passages) of culture in both unsorted, positive, and negative subpopulations. On the other side of the coin, researchers also reported the contamination and growing numbers of positive cells in the negative fractions in SSEA-4 [34] and Sca-1 [41] studies. The scale of this problem remains unknown, as a majority of researchers do not report the purity of the received fraction, and they do not conduct the analysis for further passages after the sorting.

## 5. Mesenchymal Plasticity as a Manifestation of Stochastic Stem Cell Model

The information gathered above attempts to move towards an understanding of MSC cell plasticity, but this concept itself remains difficult to explain due to numerous factors that remain vaguely explored. In 1985, Ogawa et al. [165] published a very important experiment, establishing different types of colonies arising from single cells. He proved that two daughter cells can produce completely different lineages within one cell cycle. In 2006, Zipori [166] indicated that the cells tend to change phenotypes even when obtained as clonal populations. Not only phenotype of that cells changed but also properties. Enzyme expression was different among individual cells of the same clone; adipogenesis was an inducible and reversible feature in all of the clones, the same as the capacity to support hemopoiesis. Thus, the authors concluded that mesenchymal cell lines phenotype is very flexible and environmental-dependent. The observed heterogeneity and the presence of a small population of cells with stem potential may be due to the presence of many cell types with different origins. It could also be the possibility that these stem cells are direct descendants that distribute to various organs and tissues during ontogeny and remain there throughout the mammalian life span. Alternatively, these different phenotypes may be derived from a common one universal stem cell. The latter hypothesis was supported by experiments of several research groups. Pittenger et al. [167] proved that cells forming a clone derived from a single cell can differentiate into adipocytes, chondrogenic cells, and osteocytes, confirming the multipotency of mesenchymal cells. Jiang et al. [168, 169] proved the existence of the same population of pluripotent cells in the brain and muscles, calling these cells multipotent adult progenitor cells (MAPCs). At the single-cell level, researchers confirmed the differentiation of MAPCs *in vitro* to derivatives of all three germ layers. Moreover, after implantation into the mouse blastocyst, these cells contributed to the formation of many somatic cells [170]. Such cells were found not only in bone marrow but also in muscles and brain. Zipori [166] suggested that stemness is a state that, theoretically, any cell may enter, and stemness is an unstable state characterized by promiscuous gene expression that puts the cell in a standby state, ready to commit to a variety of different directions. A new concept for the traditional stem cell model, assuming the stem cell to be the origin of an irreversible hierarchy of descending potency for renewal (deterministic model) as opposed to the stem cell state notion in which cells may assume a stem state even when already in a differentiating stage (stochastic model) due to plastic process of epigenetic remodeling.

In 2022, Quesenberry et al. [23] brought the described hypothesis much closer. The presented theory of a “universal stem cell” denies the old, deterministic model in which the fate of the cells is hierarchical and directed. The theory of universal stem cell convinces that differentiation is not ultimately determined into a given cell type, but it occurs as continuum during the cell cycle and depends on numerous factors such as the surrounding environment, paracrine factors, and more general, e.g., sex, disease status, race, or drug therapy. This model explains the phenotypic changes of cells depending on the point in the cell cycle. Possibly, further discoveries on the topic of possessing the genuine stem cell subpopulation change the perspective on the currently used stem cell classification.

## 6. Further Perspectives

In this review, we have collected available information for markers indicating distinct subpopulations within MSCs. To our surprise, reports differ between research groups, especially in calculations of positive cells expressing described markers. Discrepancies could be a result of different protocols between research groups, which emphasizes the importance of standardization of MSCs studies to minimize variations and make research more comparable.

**Figure 3:**
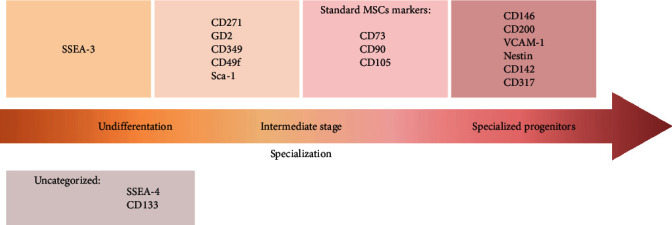
Markers described in the review in a context of cell specialization.

We presented that some of the markers could indicate the population of genuine stem cells within MSCs, while others could predict a more specialized pool of cells that potentially could be applied in wound healing or to suppress inflammatory (Figure 3). However, more studies are still needed to support those observations, especially as some markers are barely described for human MSCs like CD106 (VCAM), GD2, or CD49F; some markers, like CD349, were only described in MSCs from one tissue. For some of the markers described, there are insufficient data (CD133) or conflicting data (SSEA-4) to properly categorize them. There is also still a lack of studies linking the described subpopulations to a specific developmental origin. Most evidence is collected for the SSEA family and CD271, whereas this topic remains almost unexplored for Sca-1, GD-2, CD49F, and other markers. However, presented initial attempts linked unique properties with an undifferentiated character of described subpopulations. For more precise competitive analysis, there is a need for publishing also, so-called negative data, which is difficult in academic environment where publications showing statistically significant differences are promoted while the others are rejected. The change of perspective for academic publishing could ultimately provide the evidences for the acceptance or rejection of markers for further therapies.

Last but not least, the majority of presented subpopulations are studied only at *in vitro* level. This is understandable, as there is a need for detailed characteristics of those cells. Several subpopulations were also assessed *in vivo*, but still, there are more research to conclude. To our knowledge, only SSEA-3+ subpopulation was translated to patient studies, and currently, phase I studies are conducted. Taken together, further research on the described subpopulations is essential to progress toward the use of a homogeneous subpopulation that would be focused on treating a specific need rather than using heterogeneous MSCs that currently pose as a therapeutic standard.

## Figures and Tables

**Table 1:** Expression of markers for potential stem cells in different MSC sources.

Marker	MSCs source	% of cells	Comments	Reference
CD49f	BM	(1) 66.5 (2) 11	(1) Fetal BM (2) Adult BM -Loss CD49f expression during culture -Differences between donors	[25]

CD49f	BM	22.2%	-Passage 1 -Loss during culture passages -Differences between sources	[26]

CD49f	DM	6.68	-Passage 0 -Together with CD146 high expression	[27]

CD49f	(1) mouse AT (2) rat AT	(1): 17.7 (2): 27.2	-Passage 2 -CD49f expression decreased after TNF and IFN treatment -CD49f expression decreased with passage number	[28]

CD133	AT	12		[29]

CD133	BM	15		[29]

CD133	WJ	Less than 2		[29]

CD271	AFC	Less than 0.5		[30]

CD271	AT	8.4		[30]

CD271	AT	5		[29]

CD271	BM	(1) 22.3 (2) 10.1	(1) Young donors (2) Elderly donors	[31]

CD271	BM	3.7		[30]

CD271	BM	8		[29]

CD271	BM	1.9	Organism: mouse	[32]

CD271	CB	Less than 0.5		[30]

CD271	DM	5.5		[33]

CD271	PVC	Less than 0.5		[30]

CD271	WJ	Less than 0.5		[30]

CD271	WJ	Less than 1		[34]

CD271	WJ	2		[29]

CD349	PL	20–58	-Site of isolation: chorion laeve tissue -Expression differences between clones -Loss of MSC markers -Together with negative CD271 expression	[35]

CD349	PL	0.2	-Site of isolation not specified, -Together with Nanog, Oct4 and SSEA4 upregulation	[36]

GD2	BM, adult	(1) ∼55 (2) ∼35	(1) Passage 2, cell culture in AB-HS (2) Passage 2, cell culture in FBS	[37]

GD2	AT	46.7	–	[38]

GD2	BM	95	Passage 2, cells CD45^−^ CD105^+^CD73^+^, maintained expression for 8 passages	[39]

GD2	BM, fetal	(1) ∼88 (2) ∼65	(1) Passage 2, cell culture in AB-HS (2) Passage 2, cell culture in FBS	[37]

GD2	BM	63.4–73.9	-Organism: mouse, -Passage 2, expression differed between mouse strains	[40]

GD2	UC	(1) ∼38 (2) ∼18	(1) Passage 2, cell culture with AB-HS (2) Passage 2, cell culture in FBS	[37]

Sca-1	BM	(1) 4 (2) 0.5	(1) Compact bone marrow (2) Flushed bone marrow	[41]

SSEA-3	Mouse AT	6.3		[42]

SSEA-3	AT	3.2		[43]

SSEA-3	AT	8.8		[21]
SSEA-3	AT	1.9		[44]

SSEA-3	BM	5.3		[45]

SSEA-3	WJ (explant)	(1) 6.7 (2) 6.1	(1) Explant isolation, passage 0 (2) Enzymatic isolation, passage 0	[34]

SSEA-4	AT	10		[29]

SSEA-4	BM	(1) 5.2 (2) 4	(1) Young donors (2) Elderly donors	[31]

SSEA-4	BM	(1) 72 (2) 79.8	(1) Females (2) Males	[46]

SSEA-4	BM	55		[29]

SSEA-4	DM	5.6		[33]

SSEA-4	WJ	(1) 32.4 (2) 26.1	(1) Explant isolation, passage 0 (2) Enzymatic isolation, passage 0	[34]

SSEA-4	WJ	60		[29]

SSEA-4	WJ	(1) 35% (2) 70%–74%	Used different human platelet lysates: (1) with lower concentration of factors, (2) with higher concentration of factors.	In submission

*Note*: ∼: approximately, AB-HS: human AB serum, AFC: amniotic fluid, AT: adipose tissue, BM: bone marrow, CB: cord blood, DM: dermis, FBS: fetal bovine serum, IFN: interferon, PL: placenta, PVC: perivascular compartment of umbilical cord, TNF: tumor necrosis factor, UC: umbilical cord, WJ: Wharton Jelly. *Source of MSCs: human*, *unless otherwise stated*.

**Table 2:** Properties of subpopulations separated from MSC tissues.

Marker	Sorting method	Effect	Reference
CD49f	FACS	↑ Clonogenity, ↑ proliferation, ↑ migration, ↑ multilineage differentiation,	[26]

CD49f	FACS	↑ Colony forming, ↑ adipogenic and osteogenic differentiation	[25]

CD49f	FASC	↑ Self-renewal, ↑ spheres formation, ↑ adipogenic and osteogenic differentiation	[27]

CD49f	MASC	↑ Adhesion, ↑ proliferation, ↑ adipogenic and osteogenic differentiation, ↑ migration, ↑ antiapoptotic potential	[28]

CD271	FACS	↑ Clonogenicity, ↑ osteogenic differentiation	[30]

CD271	FACS	↑ Proliferation,	[31]

CD271	FACS	↑ Proliferation, ↓ osteogenic and adipose differentiation	[47]

CD271	MACS	↑ Neuronal and glial differentiation, ↑ migration toward islets and islet-like cell clusters	[32]

CD271	MACS	↑ Pluripotent genes expression	[48]

CD271	MACS	No changes in multipotent differentiation *in vitro*, ↑ osteochondral repair *in vivo*, ↓ angiogenesis *in vitro* and *in vivo*	[49]

CD271	MACS	↑ Adipogenesis, chondrogenesis, osteogenesis	[33]

CD349	FACS	↓ Angiogenic-properties, ↓ vasculogenesis, ↓ re-endothelialization, similar osteogenic differentiation potential	[35]

CD349	FASC	↑ Clonogenicity, ↑ multi-lineage differentiation	[36]

GD2	FACS	↑ Clonogenicity, ↑ proliferation, ↑ adipogenesis, ↑ osteogenesis, ↑ gene expression: LPL, adipsin, collagen I, CBFA1, OC, ↑ content of positive cells: Sca-1+, CD105+, SSEA-1+, Nanog+, ↓ content of positive cells: CD34+, C-kit+, CD45+, CD11b+	[40]

GD2	MACS	↑ Clonogenicity, ↑ colony size, ↑ proliferation, ↑ gene expression: SSEA-4, Oct-4, Sox-2, Nanog, Nestin, GFAP, NSE, ↑ adipogenesis, ↑ osteogenesis	[37]

Sca-1	FACS	↑ Clonogenicity, ↑ gene expression: NANOG, TERT, BMP2, Myf5, ↓ chondrogenesis, ↓ gene expression: Col2a1	[41]

Sca-1	FACS	↑ Clonogenicity	[50]

Sca-1	FCAS	↑ Gene expression: Eng (CD105), ↓ gene expression: IL-6, Pdgfra, Ly6a, Itgb1, Itga5, CD44, Thy1(CD90)	[51]

SSEA-3	FACS	↑ Differentiation into insulin-producing cells, ↑ pluripotent genes expression	[43]

SSEA-3	FACS	↑ Migration toward injured liver cells, ↑ restoration of liver function in liver fibrosis model, ↑ *in vivo* spontaneous differentiation into hepatocyte	[52]

SSEA-3	FACS	↑ Sphere formation	[22]

SSEA-3	FACS	↑ Sphere formation, ↑ pluripotency, ↑ spontaneous expression and expression after differentiation of markers from three germ layers	[21]

SSEA-3	FACS	Better biodistribution, ↑ motor and cognitive functions of HIE rats	[45]

SSEA-3	FACS, MACS	Similar effects observed in both sorting methods ↑ sphere formation, ↑ pluripotency, ↑ neural differentiation	[42]

SSEA-3	MACS	↓ Apoptosis and senescence after UV or H_2_O_2_ treatment, ↑ activation of damage repair system of DNA via non-homologous end joining	[53]

SSEA-3	MACS	↑ Pluripotent genes expression	[48]

SSEA-3	MACS	↑ Expression and secretion of growth factors, ↑ pluripotency gene expression, ↑ increased wound healing of skin ulcers	[44]

SSEA-4	FACS	No differences in proliferation, pluripotency genes expression, osteogenic differentiation, adipogenic differentiation	[34]

SSEA-4	FACS	↑ Clonogenicity, ↑ proliferation	[54]

SSEA-4	FACS	↑ Pluripotency and neural gene expression, ↑ viability of spheres, smaller spheres formed, no differences in proliferation and clonogenicity	In submission

SSEA-4	MACS	↑ Adipogenesis	[33]

SSEA-4 CD271	FACS	↑ Proliferation	[31]

SSEA-4 CD271	FACS	↑ Clonogenicity, ↓ adipogenesis	[55]

*Note*: FACS: fluorescence-activated cell sorting, MACS: magnetic activated cell sorting, HIE: hypoxic-ischemic encephalopathy.

**Table 3:** Expression of markers for more specialized subpopulations in different MSC sources.

Marker	MSC source	% of cells	Comments	Reference
CD142	WJ (explant)	71.2–88	Depending on donors	[109]

CD146	AT	5		[29]

CD146	AT	∼5		[29]

CD146	AT	∼18.9	Semiconfluent culture	[110]

CD146	BM	∼5		[29]

CD146	BM	50.1		[111]

CD146	DM	22		[33]

CD146	DP	38.8		[112]

CD146	WJ	21.8		[29]

CD200	AT	(1) 24% (2) 80%	(1) Subcutaneous fat derived cells (2) Visceral fat derived cells	[113]

CD200	BM	23–63.4	The highest level of CD200 at the density 30 × 10^3^ cells/cm^2^	[114]

CD200	PL	(1) ∼70 (2) ∼1.8	Passage 5, (1) fetal PL, (2) maternal PL	[115]

CD317	BM	1–3		[116]

CD317	BM	(1) 28 (2) 1.7	*Two distinct subpopulations: CD317dim* (*1*) *and CD317bright* (*2*)	[117]

Nestin	Kidney	1.1	Nestin-GFP mice	[118]

Nestin	Spleen	0.7	Nestin-GFP mice	[119]

VCAM	AT	0.73	Passage 3	[120]

VCAM	BM	32.04	Passage 3	[120]

VCAM	CV	65.01	Passage 3	[120]

VCAM	CV	62.9		[121]

VCAM	UC	7.44	Passage 3	[120]

VCAM	UC	88	After proinflammatory induction	[122]

*Note*: ∼: approximately, AT: adipose tissue, BM: bone marrow, CV: chorionic villi, GFP: Green Fluorescence Protein, PL: placenta, UC: umbilical cord, WJ: Wharton Jelly. *Source of MSCs: human*, *unless otherwise stated*.

**Table 4:** Properties of subpopulations separated from MSC tissues.

Marker	Sorting method	Effect	Reference
CD142	FACS	↑ Gene expression: SPARC, COL4A1, COL1A1, and COL5A1, ↑ “wound healing” potential, ↓ gene expression: MKI67, ↓ proliferation,	[109]

CD146		↑ Proliferation, ↑ immunomodulation, ↑ multilineage differentiation,	[123]

CD146	FACS	No differences in clonogenicity, proliferation, multilineage differentiation, ↑ differentiation in vascular smooth muscle cell	[124]

CD146	FACS	↑ Immunomodulation, ↑ telomere length	[125]

CD146	FACS	↑ Migration towards intervertebral discs, ↑ discogenic differentiation *in vitro*	[126]

CD146	MACS	↑ Adipogenesis, ↑ angiogenesis	[110]

CD146	MACS	↑ Differentiation, ↑ mineralization	[112]

CD200	MoFlo (Dako, Glostrup, Denmark)	↑ Level of *α*SM-actin protein, ↑ expression of *RUNX2* and *DLX5*, ↑ osteoblastic differentiation	[114]

CD317	FACS	No changes in clonogenicity, smaller cells, ↑ increased IL-7 expression and secretion	[116]

CD317	FACS	↑ Expression of CD54, CXCL10, CXCL11 and CCL2, induction of Th1 phenotype, induction of cutaneous tissue damage of skin explant model of inflammation, no tissue formation when applied in scaffold in immunocompromised mice	[117]

VCAM	EasySep (magnetic separation)	↑ Effective to modulate T helper subsets, ↓ clonogenecity	[120]

VCAM	FACS	↑ Angiogenic potential	[121]

*Note*: FACS: fluorescence-activated cell sorting, IL: interleukin, MACS: magnetic-activated cell sorting.

## Data Availability

No underlying data were collected or produced in this study.

## Abbreviations

AD-MSCs: Adipose tissue-derived mesenchymal stem/stromal cells
ANG: Angiogenin
ANGPT2: Angiopoietin 2
BDNF: Brain derived neurotrophic factor
BM-MSCs: Bone marrow-derived mesenchymal stem/stromal cells
CFU: Colony forming unit
CIA: Collagen-induced arthritis
CNS: Central nervous system
CV: Placenta chorionic villi
CXCL1: C-X-C motif chemokine ligand 1
DP: Dental pulp
EAE: Experimental autoimmune encephalomyelitis
ECM: Extracellular matrix
EMT: Epithelial-to-mesenchymal transition
ESCs: Embryonic stem cells
FACS: Fluorescence-activated cell sorting
FGF: Fibroblast growth factor
FZD-9: Frizzled-9
GD2: Neural ganglioside
GFP: Green fluorescence protein
HGF: Hepatocyte growth factor
IFN*γ*: Interferon gamma
IL-1*β*: Interleukin 1 beta
IL-8: Interleukin 8
IPSCs: Induced pluripotent stem cells
IVD: Intervertebrate discs
ISCT: International Society of Cell & Gene Therapy
HSCs: Hematopoietic stem cells
LPM: Lateral plate mesoderm
MACS: Magnetic-activated cell sorting
MAPCs: Multipotent adult progenitor cells
MCAM: Melanoma cell adhesion molecule
MET: Mesenchymal-to-epithelial transition
MSCs: Mesenchymal stem/stromal cells
MUSE Cells: Multi-lineage differentiating stress enduring cells
NCSCs: Neural crest-derived stem cells
NGF: Nerve growth factor
NSCs: Neural Stem Cells
PL-MSCs: Placenta-derived mesenchymal stem/stromal cells
PNS: Peripheral nervous system
Sca-1+: Stem cells antigen-1
SSEA: Stage-specific embryonic antigen
TNF*α*: Tumor necrosis factor alpha
UC-MSCs: Umbilical cord-derived mesenchymal stem/stromal cells
VCAM- 1: Vascular cell adhesion molecule 1
WJ-MSCs: Wharton jelly-derived mesenchymal stem/stromal cells.
